# DFT Investigation of the Structural, Electronic, and Optical Properties of AsTi (B*_i_*)-Phase ZnO under Pressure for Optoelectronic Applications

**DOI:** 10.3390/ma16216981

**Published:** 2023-10-31

**Authors:** Muhammad Adnan, Qingbo Wang, Najamuddin Sohu, Shiyu Du, Heming He, Zhenbo Peng, Zhen Liu, Xiaohong Zhang, Chengying Bai

**Affiliations:** 1 Engineering Laboratory of Advance Energy Materials, Ningbo Institute of Materials Technology and Engineering, Chinese Academy of Sciences, Ningbo 315201, China; adnanphy_53@hotmail.com; 2 University of Chinese Academy of Sciences, 19A Yuquan Rd., Shijingshan District, Beijing 100049, China; 3 GC University Hyderabad, Sindh 71000, Pakistan; najam_sohu@yahoo.com; 4 School of Mathematics and Physics, China University of Geosciences (Wuhan), Wuhan 430074, China; qingbowang2013@163.com; 5 School of Materials Science and Engineering, China University of Petroleum (East China), Qingdao 266580, China; 6 Milky-Way Sustainable Energy Ltd., Zhuhai 519099, China; hilltopper@163.com; 7 Yangtze Delta Region Institute of the University of Electronic Science and Technology of China, Huzhou 314299, China; 8 Institute of Energy Storage & Conversion Technology, Ningbo Polytechnic, Ningbo 315800, China; zpeng@nbpt.edu.cn; 9 Key Laboratory of Superlight Materials and Surface Technology, Ministry of Education, College of Materials Science and Chemical Engineering, Harbin Engineering University, Harbin 150001, China; tcliu1989@163.com (Z.L.); zhangxiaohong@hrbeu.edu.cn (X.Z.); chengyingbai@hrbeu.edu.cn (C.B.)

**Keywords:** B*_i_*-phase ZnO, first-principles method, B3LYP, optoelectronic properties

## Abstract

Pressure-induced phases of ZnO have attracted considerable attention owing to their excellent electronic and optical properties. This study provides a vital insight into the electronic structure, optical characteristics, and structural properties of the AsTi (B*_i_*) phase of ZnO under high pressure via the DFT-based first-principles approach. The phase transformation from BN(B*_k_*) to the B*_i_* phase of ZnO is estimated at 16.1 GPa using local density approximation, whereas the properties are explored precisely by the hybrid functional B3LYP. The electronic structure exploration confirms that the B*_i_* phase is an insulator with a wider direct bandgap, which expands by increasing pressure. The dielectric function evidenced that the B*_i_* phase behaves as a dielectric in the visible region and a metallic material at 18 eV. Optical features such as the refractive index and loss function revealed the transparent nature of the B*_i_* phase in the UV range. Moreover, the considered B*_i_* phase is found to possess a high absorption coefficient in the ultraviolet region. This research provides strong theoretical support for the development of B*_i_*-phase ZnO-based optoelectronic and photovoltaic devices.

## 1. Introduction

Semiconductor oxides, with a wider bandgap, large exciton energy, and outstanding optical properties, are supposed to be prominent candidates in developing optoelectronic and photovoltaic devices [1,2,3]. ZnO is one of the most efficient semiconductor oxide materials found as a zincite mineral in wurtzite form, and it possesses a hexagonal crystal arrangement [4,5]. The ZnO (bandgap 3.4 eV) has been extensively studied recently due to its industrial applications, such as in LEDs [6,7,8,9], biosensors [10,11,12,13], photodetectors [14,15], and solar cells [16,17]. Modern computational techniques [18,19] and experimental high-pressure equipment [20] have enabled researchers to achieve a transition in the wurtzite ZnO structure towards new phases accompanying extraordinary properties under pressure.

Wurtzite B4 (stable-phase) ZnO has experimentally been found to transform into the rock salt (B1)-type structure at a high pressure of 9.1 GPA, and it has been analyzed for properties using X-ray diffraction and Mossbauer spectroscopy [21,22,23]. This transition is taken as a basic reference in ongoing research on the pressure-induced phases of ZnO. Some researchers, using first-principles computational techniques, have also found a direct transition route of the B4 phase to rock salt NaCl-type B1, cesium chloride CsCl-type B2, zinc blende B3, and lead oxide PbO-type ZnO under high pressure [24,25,26,27,28,29]. Additionally, the intermediary phases between these routes, such as the GeP phase [30], tungsten carbide WC (B*_h_*) phase [31], NiAs (B81) phase [32], and BN(B*_k_*) phase [33], are also observed theoretically under high pressure. Moreover, ZnO is also theoretically investigated for transitions and properties under low pressure [34], and the metastable *β*-BeO phase is achieved at a negative pressure of −10.2 GPA [35]. 

Nevertheless, the search for new phases of ZnO with excellent properties is still a continuing research interest for modern-day applications. Recently, Molepo et al., using ab initio simulations, predicted an arsenic titanium AsTi (B*_i_*)-type structure in the ZnO system, with exceptional properties. The B*_i_*-type ZnO is proposed to act as an intermediate phase in the conversion route B4→B*_k_*→B*_i_*→B2 phase at about 16.1 GPa [36]. The literature review has confirmed that the novel properties of Bi-phase ZnO have not yet been explored under variable high pressure. Hence, it is essential to explore the structural characteristics, electronic structure, and optical characteristics of Bi-phase ZnO under stress, which may be useful in developing numerous industry-required devices [37,38].

The bandgap affects a material’s properties significantly; for instance, outstanding optoelectronic properties of semiconductor oxides and perovskites result from large bandgaps [39]. The density functional theory (DFT)-based first-principles method is recognized as an efficient computational approach for examining the structural phase transition as well as the properties of materials under different pressure and temperature conditions [40,41,42,43]. However, the literature survey has indicated that the LDA and GGA first-principles approximations generally underestimate the bandgap energy of metal oxides like ZnO. However, according to research results, the Becke three-parameter Lee–Yang–Parr functional (B3LYP), as a hybrid functional, is found to be an effective method for computing the band structure and optical properties with sufficient accuracy [44,45,46,47,48].

Based on the above rationale, we attempt to extensively investigate the behavior, structural dynamics, electronic states, and optical characteristics of Bi-phase ZnO with respect to varying pressure conditions. The first-principles LDA technique is employed successfully to find the structural transition from the BN(B*_k_*) phase to the AsTi (B*_i_*) phase of ZnO. Inspection of the structural characteristics of the B*_i_* phase is then carried out to see whether it would remain stable under high pressure. The hybrid functional method has enabled the accurate estimation of the bandgap energy and optical characteristics of the B*_i_* phase. The acquired results predict the distinctive insulator, dielectric and metallic behavior, and transparent properties of the B*_i_* phase under different applied pressures. This research provides theoretical guidance for designing ZnO-based photodetectors, nanolasers, and photovoltaic solar cells [14,15,16,17].

## 2. Computational Details

**Figure 1:**
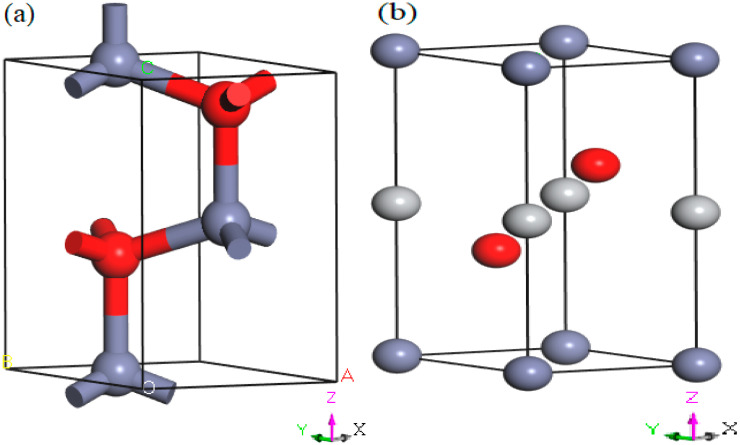
The optimized crystal structures of the B*_k_* (**a**) and B*_i_* phase (**b**) at 0 GPa. The red-colored balls denote atoms of oxygen and The gray balls represent Titanium atoms and blue balls represent Zinc atoms.

The Cambridge Sequential Total Energy Package (CASTEP) Program inbuilt into material studio software was employed to perform DFT calculations [18]. The whole calculation scheme includes modeling the structure, geometry optimization, and estimation of the structural parameters, band structures, and optical aspects of B*_k_*- and Bi-phase ZnO. We employed the LDA and the Ceperley–Alder and Perdew–Zunger (CA-PZ) approach [49] for geometry optimization. For the first Brillouin zone sampling, the Monkhorst–Pack k points of 5 × 5 × 4 for the B*_k_* phase and 6 × 6 × 4 for the B*_i_* phase were used. The cutoff value of energy was 571.40 eV, while the energy of convergence was set at 2 × 10^−5^ eV/atom. In the B*_k_* and B*_i_* phases, zinc and oxygen atoms were in the electronic configurations 3d^10^4s^2^ and 2s^2^2p^4^, respectively. To deal with interactions among electrons, we used the ultrasoft pseudopotentials [50,51] and Broyden–Fletcher–Goldfarb–Shanno (BFGS) algorithm [52]. After modeling the structures, geometry optimization was performed under the applied hydrostatic pressure (0–30 GPA) within the Parrinello–Rahman approach [53]. The acquired enthalpy values are graphed to find the structure transition from B*_k_*- to Bi-phase ZnO. We determined the total energy of the B*_k_* and B*_i_* structures for various volumes to show the energy–volume relation (Figure 1).

To explore the electronic states and optical properties under varying high pressure, the B3LYP functional with a separate exchange-correlation (X-C) functional value and the norm-conserving pseudopotentials were used [54]. The adopted X-C functional factor value is 0.4. The Monkhorst–Pack k points for the B*_k_* phase were fixed at 3 × 3 × 2 and 4 × 4 × 2 for the B*_i_* phase. The cutoff energy in these calculations was kept at 1142.90 eV, which infers that the plane wave in our computations has kinetic energy (*ħ*^2^*k*^2^/2 m) under 1142.90 eV. With our selected cutoff energy, we could ensure precision and convergence while requiring minimal computational costs. Throughout this work, all calculations were carried out in reciprocal space. The bandgap energy estimated for the B*_k_* phase is 3.46 eV, which is in good agreement with previous research [33]. This result validates the accuracy of the computational parameters and methodology used in our research.

## 3. Results and Discussion

### 3.1. Phase Transitions and Structural Properties

**Figure 2:**
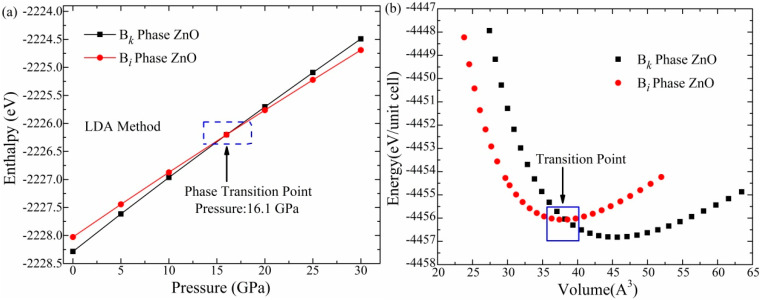
(**a**) The enthalpy against stress graph for B*_k_* and B*_i_* phase. The crossing point in the blue area is the phase conversion from the B*_k_* to the B*_i_* phase. (**b**) Energy versus volume *E*(*V*) graph for B*_k_* and B*_i_* phases of ZnO estimated at various fixed volumes.

Since unique optoelectronic properties are produced in the material as a result of the structural phase shift, the phase transition of ZnO’s B*_k_*-type structure into the B*_i_* under pressure is the foundation of this research. The LDA approach was used to perform geometry optimization in the applied pressure range of 0–30 GPa for determining the enthalpy (H) of the B*_k_* and B*_i_* phases. The computed enthalpy values were then plotted to find the transition point. The enthalpy values of B*_k_*- and Bi-phase ZnO are found to increase by increasing pressure. Figure 2a shows the intersection point where the enthalpy values of the two phases are equivalent, which demonstrates the transition of the B*_k_* phase to the B*_i_* phase. The LDA technique successfully estimated the transition at 16.1 GPa, matching well with the predicted transition pressure by Molepo et al. [36]. These results attest to the validity of the applied technique of this work.

The structures of a material demonstrate its stability as well as its influence on the electronic structure and optical behavior under different pressure conditions. Table 1 lists the computed structural characteristics, including lattice values, bond angles, and bond lengths, for the B*_k_* and B*_i_* phases at 0 GPa and the transition pressure at 16.1 GPa. The lattice constant values are found to decrease constantly with increasing pressure. The lattice constants of the B*_i_* phase at 0 GPa were estimated as a = b = 2.920 Å, c = 5.024 Å, which decreased to a = b = 2.851 Å, c = 4.949 Å at a transition pressure of 16.1 GPa. Additionally, the cell volume at 0 GPa was found to be 37.114 Å^3^, which decreased to 34.842 Å^3^ at 16.1 GPa. The hybridization between Zn and O bonds is ultimately influenced by the bond length, which steadily decreased from 2.102 Å at 0 GPa to 2.059 Å at 16.1 GPa. Moreover, the findings showed that pressure did not affect the bond angle of the B*_i_* phase, with α = β = 90° and γ = 120° at both 0 GPa and 16.1 GPa.

The relationship between energy and volume was also investigated, and the resulting data are presented in Figure 2b. According to the results, B*_k_*- and Bi-phase ZnO retain low energy at equilibrium points. Additionally, the graph demonstrates that the B*_i_* phase’s minimum energy value is higher than the B*_k_* phase’s lowest energy value. Hence, the B*_k_* phase is observed to be a more stable structure at ambient conditions. The cross point of the energy–volume graph indicates the equivalent energy of phases at this point, and the phase transition happens at this particular point. These findings contribute to understanding the behavior and stability of the B*_i_* phase for developing nano-structured devices i.e., ZnO nanolasers, with tailored efficiency under pressure [55].

### 3.2. Bandgap and Structure

**Figure 3:**
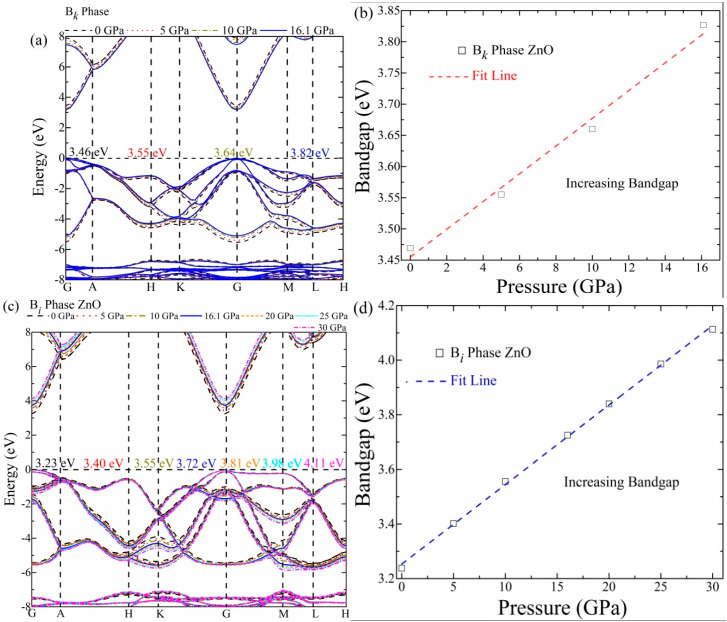
(**a**) The estimated electronic structure of B*_k_* phase under 0~16.1 GPa, (**b**) The band energy values against the stress graph for B*_k_*-phase ZnO, (**c**) Band structure of B*_i_* phase at (0, 5, 10, 16.1, 20, 25 and 30 GPa), (**d**) Band energy versus stress graph for Bi-phase ZnO.

The bandgap is an important intrinsic property of a material that determines properties such as electrical conductivity and optical absorption for optoelectronic and photovoltaic applications. We applied the hybrid functional (B3LYP) approach to precisely analyze the band, to deal with the structure of optimized B*_k_*- and Bi-phase ZnO. Figure 3a shows the B*_k_*-phase band structure for the energy range of −8~8 eV under the high-pressure range of 0–16.1 GPa. It is found that the conduction band minimum (CBM) and valence band maximum (VBM) are located at the same point, so the B*_k_* phase possesses a direct bandgap. The calculated bandgap of the B*_k_* phase is 3.467 eV, as shown in Figure 3a, which is in close agreement with the previous computational results [33], demonstrating the validity of our applied technique and the results acquired. With an increase in applied pressure, the bandgap is seen to expand progressively. The bandgap of the B*_k_* phase is found to increase to 3.555, 3.647, and 3.827 eV at 5, 10, and 16.1 GPa, respectively, with the latter being the structure conversion pressure. Figure 3b demonstrates the direct relation between bandgap and applied pressure (GPa), shown by a fitted linear graph. The expected band energy value and the trend of increasing bandgap with increasing stress are consistent with those found in the earlier literature [33]. The conduction state advances to a higher energy state due to an increasing repulsion between Zn and O atoms due to changes in bond length and the resulting repulsion of the electronic cloud. Furthermore, as the Zn-O hybridization is intensified, the valence state is moved to a state of lower energy.

The band structure results (Figure 3b) for the B*_i_* phase of ZnO under increasing pressure between 0 and 30 GPa are also plotted. The B*_i_* phase is expected to have a direct band energy at “0” GPa. The B*_i_* phase is revealed to have a high bandgap energy value of 3.23 eV, which indicates that it may be active at high temperatures and in the UV range. The bandgap is observed to gradually increase to 3.40, 3.55, 3.72, 3.81, 3.98, and 4.11 eV with an increasing pressure of 5, 10, 16.1, 20, 25, and 30 GPa, respectively. The results confirm that the bandgap progressively expands with increasing pressure. The large bandgap energy infers that the B*_i_* phase nearly possesses an insulator nature. The change in electronic characteristics may result in inducing a shift in the optical properties. The linear relation between bandgap and pressure (GPa) is expressed by a linear graph in Figure 3d.

### 3.3. Electronic Density of States

**Figure 4:**
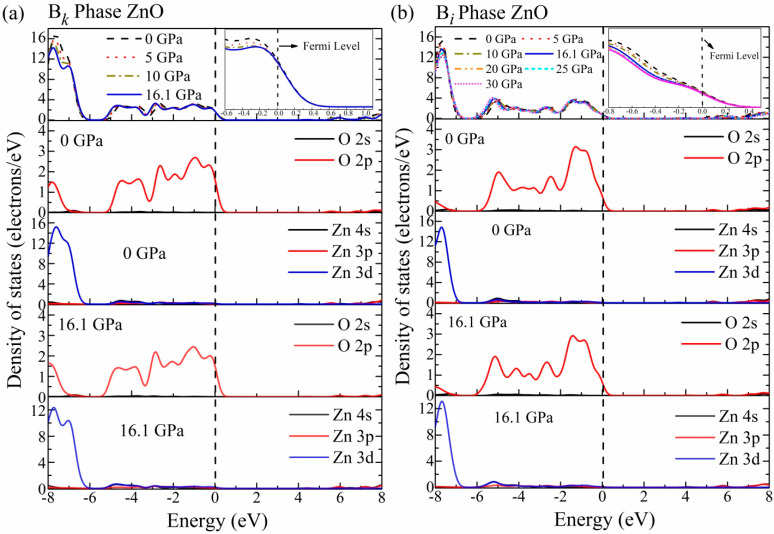
The estimated total and partial density of states (DOS) of (**a**) B*_k_*-phase ZnO under 0~16.1 GPa, (**b**) B*_i_* phase at (0~30 GPa). The perpendicular line at “0” displays the Fermi level b/w the valence and conduction bands.

The electronic density of states (DOS) is a significant aspect that defines the electronic and optical characteristics of a material. To assess the role of various electronic states in optical characteristics, we also estimated the total density of states (TDOS) and partial DOS (PDOS). Figure 4 displays the obtained results for the B*_k_* phase in the pressure range of 0–16.1 GPa and the B*_i_* phase under 0–30 GPa. For this computation, the Fermi level was adjusted to 0 eV. The energy levels of −6.2 and −6 eV distinguish the values of the valence state for the B*_k_* structure at 0 and 16.1 GPa, respectively. It has been noted that the pressure has an impact on hybridization, and because of the repulsion of particular electron states of zinc and oxygen atoms that are responsible for bonding, the resulting DOS is expended. Additionally, the shift of the conduction band to a higher energy state and the valence band to a low energy state can be observed. This is consistent with the band structure calculations. With the increase in peak value as well as the shift and change in the shape of the DOS profile, a rise in intriguing optical features is clearly observed. 

The DOS and PDOS acquired by the B*_i_* phase under increasing pressure are discussed here in detail and shown in Figure 4b. According to the graph, the energy values for the valence bands at 0 GPa and 16.1 GPa for the B*_i_* phase are −6.8 and −6.6 eV, respectively. The valence band energy of 0~−9.4 eV is produced due to the combination of two energy regions. The bottom zone (−9.4~−6.8 eV) comes from the Zn 3d energy state, whereas the higher region (−6.8~0 eV) is associated with the O 2p electronic state. The O 2s, O 2p, Zn 4s, and Zn 3p electronic states contribute to the energy of the conduction band. At a transition pressure of 16.1 GPa, the graph shows that the valence band of the energy range of −9.7~0 eV is divided into two energy ranges. The energy range between −9.7 and −6.6 corresponds to the Zn 3d state, whereas the energy area between −6.6 and 0 corresponds to the O 2p electronic state. The conduction bands are mostly constituted from the O 2p, Zn 4s, and Zn 3p states. Strong Zn 3d and O 2p state hybridization leads to the formation of the zinc–oxygen bond, which indicates that the B*_i_* phase has a covalent character.

### 3.4. Optical Properties

**Figure 5:**
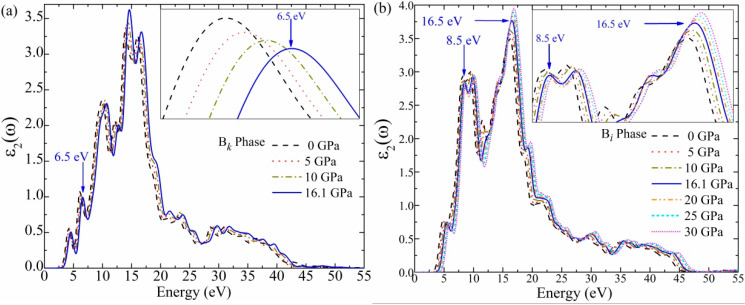

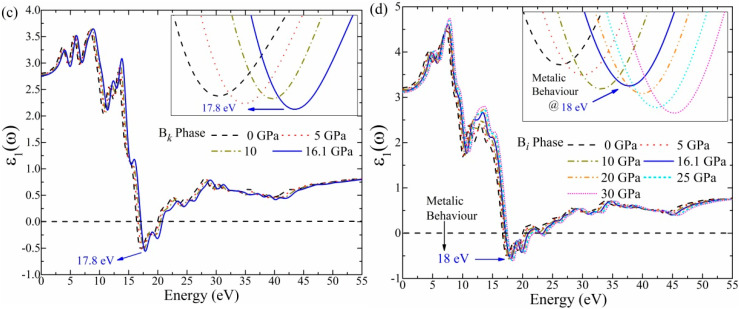
The calculated imaginary part *ε*_2_(*ω*) (**a**,**b**) and real part *ε*_1_(*ω*) (**c**,**d**) of the dielectric function for B*_k_* (0~16.1 GPa) and B*_i_* phases (0~30 GPa).

**Figure 6:**
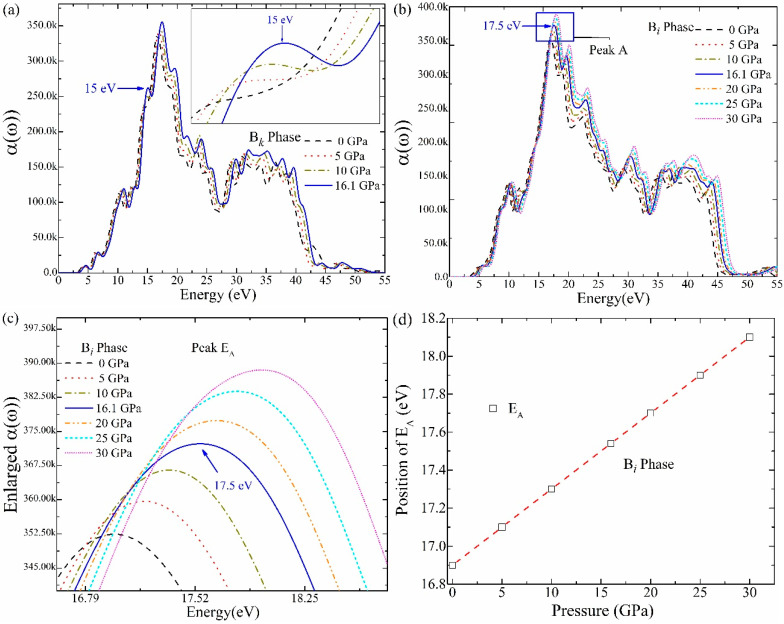

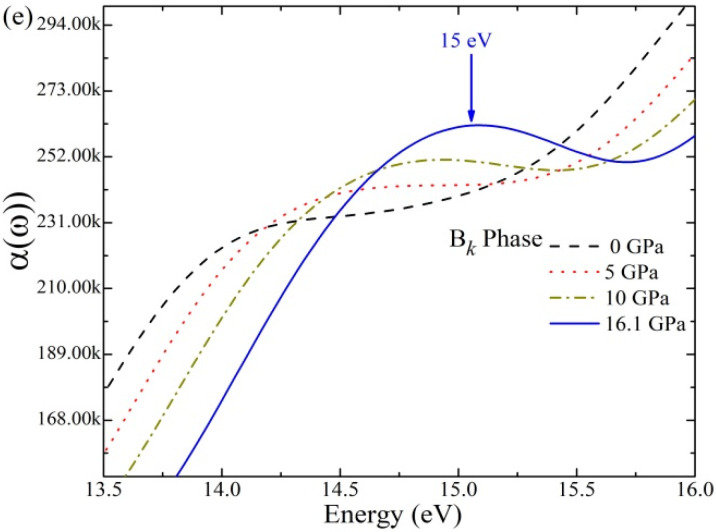
The absorption coefficient *α*(*ω*) (**a**) B*_k_* phase ZnO and (**b**) B*_i_*-phase ZnO, (**c**) magnified view of absorption for B*_i_* phase under stress 0~30 GPa, (**d**) position of *E*_A_ versus stress graph, (**e**) large view of absorption for B*_k_* phase under stress 0~16.1 GPa.

**Figure 7:**
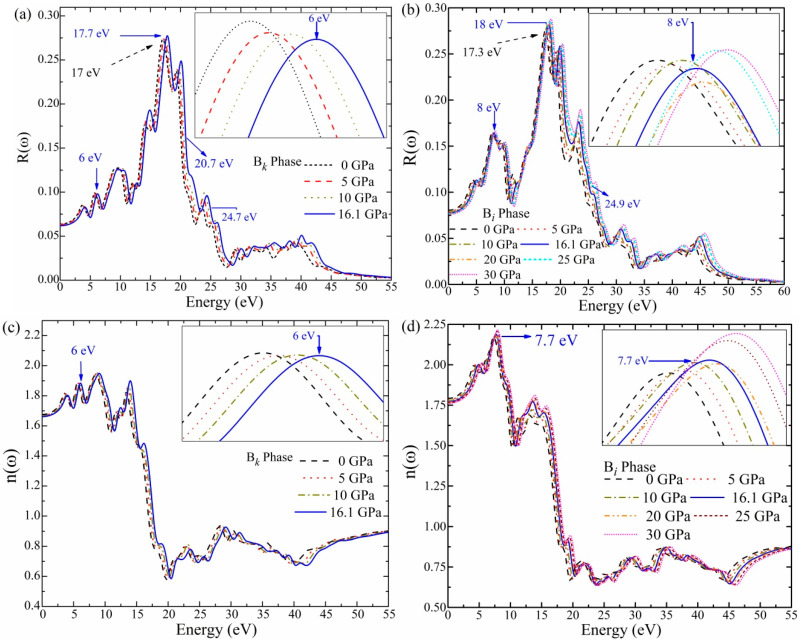
The reflectivity *R*(*ω*) (**a**,**b**), The real part of refractive index *n*(*ω*) (**c**,**d**).

**Figure 8:**
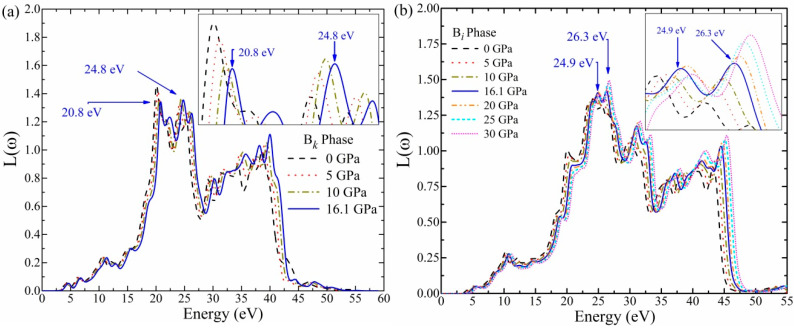
The calculated loss function *L*(*ω*) (**a**,**b**), results for B*_k_* and B*_i_* phases under 0~16.1 and 0~30 GPa.

The optical aspects of a material reveal its potential in application for optoelectric and photovoltaic devices. Hence, the precise measurement of their optical characteristics, such as reflectance *R*(*ω*), energy loss function *L*(*ω*), absorption *α*(*ω*), refractive index *n*(*ω*), and dielectric function *ε(ω*), is required. Moreover, it is also well-known that pressure can change the structure and electronic properties, which results in inducing application-oriented manipulative optical properties. Thus, the potential optical functions (Figure 5) and constants (Figure 6, Figure 7 and Figure 8) are calculated for the B*_k_* phase under the pressure range 0–16.1 and the B*_i_* phase under 0–30 GPa using the hybrid functional B3LYP at 0K.

### 3.5. Optical Function: Dielectric Function

The optical sensitivity of materials to light is described by the complex dielectric function *ε*(*ω*) represented by the equation *ε*(*ω*) = *ε*_1_(*ω*) + *iε*_2_(*ω*). The real part describes the photon dispersion, whereas the imaginary part value infers photon absorption by the electronic shift between the occupied and empty states. It is possible to compute the real part *ε*_1_(*ω*) using the Kramer–Kronig relation, while the imaginary part *ε*_2_(*ω*) is calculated using momentum matrix components between filled and unfilled levels in electronic wave functions. In order to evaluate the variation in optical characteristics due to pressure in the B*_k_*- and B*_i_*-type ZnO, the dielectric functions are calculated at different pressures in this study.

The imaginary part of the dielectric function *ε*_2_(*ω*) is calculated by means of the equation

(1) $$\varepsilon_{2}\left(\omega \right)=\frac{{2e}^{2}\pi }{\Omega \varepsilon_{0}}\begin{array}{c} \sum \\ k,v,c \end{array}|\langle \Psi_{k}^{c}|\hat{u}\cdot r|\Psi_{k}^{\nu }\rangle |\delta (E_{k}^{c}-E_{k}^{v}-E)$$

The frequency of photons that came in contact with the crystal is represented by “*ω*” in this equation. The “Ω” represents unit cell volume, “*ε*_0_” is free space permittivity, and “k” is the reciprocal lattice vector. The “*c*” and “*v*” superscripts denote the valance and conduction bands, respectively. The real part *ε*_1_(*ω*) is calculated using the Kramer–Kronig relation

(2) $$\varepsilon_{1}\left(\omega \right)=1+\frac{2}{\pi }Ρ\int_{0}^{\infty }\frac{\omega^{\prime }\varepsilon_{2}(\omega^{\prime })}{\omega^{\prime^{2}}{-\omega }^{2}}d\omega^{\prime }$$

Here, function *Ρ* is taken as the principal value.

The computed imaginary part *ε*_2_(ω) for B*_k_*- and B*_i_*-type ZnO in the energy range of 0 to 55 eV is presented in Figure 5. The initial energy value for the B*_k_* phase at 0 and transformation pressure of 16.1 GPa is found to be 2.3 and 2.9 eV, as shown in Figure 5a. The noticeable peaks are located at 4.5 and 6.5 eV at 16.1 GPa. These peaks mostly result from the optical transition between the conduction band minimum and the valence band maximum. In addition, the robust transition to the unfilled orbital of the conduction level is responsible for producing the significant peak at 14.5 eV. 

The calculated *ε*_2_(ω) results for the B*_i_* phase are shown in Figure 5b. The starting energies are 2.9 and 3.5 eV at pressures of 0 and 16.1 GPa, respectively. It can be seen that between 4.2 and 45 eV, the B*_i_* phase profile is expanded and has a higher value relative to the B*_k_* phase as a whole. The expansion in the corresponding DOS is the reason for the expansion of the *ε*_2_(ω) profile. This result should be noted for practical applications as it demonstrates that the B*_i_* phase may have excellent characteristics in the UV range. The resulting graph further demonstrates that the transition from the B*_k_* to the B*_i_* phase occurred at a clearly increased peak (8.5 eV) at 16.1 GPa. The Zn 3d transition to the O 2p level is the origin of this peak. The O 2p transition to Zn 4s is attributed to the peak at 5.5 eV. At 16.5 eV, the maximal *ε*_2_(ω) peak is found due to the Zn 3d transition to the O 2s state [56]. The wider bandgap value of 3.23–3.72 eV is responsible for the profile alteration, including the blue shift.

The real component of the dielectric function *ε*_1_(*ω*) is supposed to be the essential characteristic for illustrating the optoelectronic applications under pressure. The starting *ε*_1_(ω) for the B*_k_* phase is 2.7 eV at 0 GPa and 2.8 eV at 16.1 GPa, according to the computed values given in Figure 5c. Below 4 eV, a minimum change is observed in the *ε*_1_(ω) profile of the B*_k_* phase and nearly maintains the same shape. It is revealed that several *ε*_1_(ω) peaks occur in the negative area, mainly at 17.8 eV. Concerning *ε*_1_(ω) of the B*_i_* phase at a transition pressure of 16.1 GPa, the starting *ε*_1_(ω) is 3.1 eV, as compared to 3.2 eV at 0 GPa, as seen in Figure 5d. Below 4 eV, the B*_i_* phase shows little fluctuation in *ε*_1_(ω) value. These findings demonstrate that the pressure impact in the small energy zone is minimal. It is validated from the profile of *ε*_1_(ω) that the B*_i_* phase exhibits dielectric behavior in the visible range of energy. Further, the starting *ε*_1_(ω) energy value of 2.8 eV for the B*_k_* phase is changed to 3.1 eV for the B*_i_* phase at 16.1 GPa. This blue shift, or energy upsurge, marks the change from the B*_k_* structure to the B*_i_* structure. However, a few prominent *ε*_1_(ω) peaks fall into the negative region at 18 eV, which demonstrates the dielectric nature of the B*_i_* phase, which exhibits metallic behavior. The material’s metallic nature indicates that it has excessive charge carriers, whereas its dielectric nature designates that it is deficient in charge carriers. In general, it has been shown via dielectric functional calculation findings that the B*_i_* phase exhibits a wide range of properties and behaviors below 50 eV. The obtained results significantly express the future uses of the B*_i_* phase in the development of electronic devices.

### 3.6. Optical Constants

The optical reaction of a material to the incident light is primarily determined by the absorption coefficient (*α*(*ω*)), reflectivity (*R*(*ω*)), loss function (*L*(*ω*)), and real component of the refractive index (*n*(*ω*)). These functions also reveal the material’s important and prospective features for use in optoelectronic devices. Figure 6, Figure 7 and Figure 8 show the results of the optical constants for the B*_k_* and B*_i_* phases for a photon energy range of 0 to 55 eV.

### 3.7. Absorption Coefficient (α(ω))

The absorption coefficient is a significant property that defines the intensity attenuation of light passing through a material. The following equation, which also takes into account the dielectric function, can be used to compute the absorption coefficient.

(3) α(ω)=2ωε12ω+ε22ω−ε1ω12

According to Figure 6a, the B*_k_* phase’s absorbance coefficient *α*(*ω*) exhibits a clear peak at 6.7 eV at a transition pressure of 16.1 GPa. The observations in Figure 6b demonstrate that the *α*(*ω*) of the B*_i_* phase has a broader energy area, expressing its wide range of applications. The peak values of the B*_i_* phase undergo a blue shift as pressure increases. Compared to the highest values of the B*_k_* phase, the peak values of the B*_i_* phase are increased. The phase transition from the B*_k_* to the B*_i_* phase is epitomized well by the increased peak of the B*_i_* phase (17.5) at 16.1 GPa. When determining structure transition and figuring out the pressure after calibration, the acquired blueshift of *α*(*ω*) peaks with increasing pressure is significant. In the infrared and visible energy ranges, the absorption value of the B*_i_* phase is determined to be roughly zero. These results show that the B*_i_* phase possesses low absorption, and all the incident photons reflect from the surface. Hence, Bi-phase ZnO may be used for designing efficient devices.

The increasing band energy and the variation in the density of states are responsible for the blue shift in the absorption profile. The maximum absorption peak is found at 17.5 eV, which exists in the UV energy region. This reveals that the B*_i_*-type ZnO is a potentially viable material for ultraviolet photon absorption and should be considered for practical applications. We have chosen peak “A” of the B*_i_* phase to assess and express the structure transformation under pressure. As an illustration, the relationship between shifting peak E_A_ positions and increasing stress for the optical absorption *α*(*ω*) is explained in detail. The relationship of other optical constants could be anticipated in a similar way. Figure 6c shows the magnified view of the absorption peak “A” for the pressure-induced B*_i_* phase. It demonstrates how the peak position gradually increases as pressure increases from 0 to 30 GPa. The direct relation between *α*(*ω*) and pressure (GPa) is presented by a fitted linear graph in Figure 6d. 

#### 3.7.1. Reflectivity (*R*(*ω*))

The ratio of incident and reflected wave energy from the surface of the material is known as the reflectivity *R*(*ω*). The following equation can be used to calculate the reflectivity of a material.

(4) $$R\left(\omega \right)={\left|\frac{\sqrt{\varepsilon_{1}\left(\omega \right)+{i\varepsilon }_{2}\left(\omega \right)}-1}{\sqrt{\varepsilon_{1}\left(\omega \right)+{i\varepsilon }_{2}\left(\omega \right)}+1}\right|}^{2}$$

Figure 7 shows the reflectance *R*(*ω*) curve versus photon energy (eV) due to pressure. It is noted that the *R*(*ω*) values for the B*_k_* and B*_i_* phases are almost persistent in the infrared energy region under 1.7 eV. The B*_k_* phase shows a noticeable peak at 6 eV at a transition pressure of 16.1 GPa. For the B*_k_* phase, the maximal *R*(*ω*) peaks are observed at around 17 and 17.7 eV at 0 GPa and 16.1 GPa. Furthermore, there is a peak reduction in the *R*(*ω*) of the B*_k_* phase at 20.7 and 24.7 eV. Importantly, the transition from the B*_k_* to the B*_i_* phase is shown in Figure 7b, which indicates that the peak location of the B*_i_* phase increased to a value of 8 eV, in comparison to the 6 eV of the B*_k_* phase. In the ultraviolet range, the largest *R*(*ω*) peaks for the B*_i_* phase are found at around 17.3 eV (at 0 GPa) and 18eV (at 16.1 GPa). The measured extreme *R*(*ω*) relates to the lower region of *ε*_1_(ω). A noticeable peak drop occurred in the *R*(*ω*) of the B*_i_* phase at an energy of 24.9. The *R*(*ω*) peak drop is associated and matches well with the highest peaks found (24.9) in the loss function *L*(*ω*) calculation. For energies larger than 50 eV, it is noticed that the reflectance of the B*_i_* phase is nearly zero. These results provide a foundation for developing Bi-phase ZnO-based photovoltaic devices.

#### 3.7.2. Refractive Index (*n*(*ω*))

The refractive index *n*(*ω*) demonstrates the ability of a material to bend the light, as well as the phase velocity of a photon in that material. It is an important factor for photonic applications like optical waveguides since it assesses the material’s behavior in optical dispersion. The equation used to calculate the refractive index is given below.

(5) nω=ε12ω+ε22ω+ε1ω12/2

In Figure 7c,d, a graph is presented to show the computed refractive index *n*(*ω*) for the B*_k_* and B*_i_* phases of ZnO. It is noted that for the same pressure range, the *n*(*ω*) profile for B*_k_* and B*_i_* phases matches the *ε*_1_(ω) profile. The B*_i_* phase undergoes a blue shift and has a peak value at 7.7 eV, which is greater than the B*_k_* phase (6 eV). This blue shift in the peak value specifies a phase change from B*_k_*- to Bi-phase ZnO. A consistent peak increase in the *n*(*ω*) profile is noticed after 15.3 eV. At a transition pressure of 16.1 GPa, the *n*(*ω*) value for the B*_k_* phase begins at 1.64 eV and rapidly rises under 10 eV. The refractive index *n*(*ω*) for the B*_i_* phase is simultaneously initiated at 1.75 eV and rises steadily. At 16.1 GPa, the maximum peak of the refractive index is found at 7.7 eV. Hence, the refractive index calculation results show that the Bi-phase ZnO is a transparent material in the UV region.

#### 3.7.3. Energy Loss Function (*L*(*ω*))

The energy loss function *L*(*ω*) depicts the loss in energy of an electron passing through a material undergoing an elastic collision. It is a key factor in determining the structure and properties of a material. Equation (6) is used to calculate the energy loss function of a material. The *ε*_1_(*ω*) term in the energy loss function reveals the response of a solid to an external E.M perturbation.

(6) $$L\left(\omega \right)=\frac{\varepsilon_{2}\left(\omega \right)}{\varepsilon_{1}^{2}\left(\omega \right)+\varepsilon_{2}^{2}\left(\omega \right)}$$

The *L*(*ω*) results for the B*_k_* and B*_i_* phases are presented in Figure 8a,b. Under the energy range of 4 to 15 eV, the *L*(*ω*) peaks increase consistently, and a sudden increase is observed above this value. The phase transition is evidenced by the increase in the low peak value (20.8, 24.8 eV) of the B*_k_* phase to the higher peak value (24.9, 26.3 eV) of the B*_i_* phase at 16.1 GPa. The starting energy loss function value of the B*_i_* phase is found to be roughly zero, which should be noted for its practical applications. The prominent peaks in the loss function *L*(*ω*) graph indicate that the plasma resonance and associated frequency values are plasmon frequencies. These peaks lie in the ultraviolet region of energy. The calculated maxima peaks, i.e., 20.8 eV and 24.9 eV, of *L*(*ω*) for the B*_k_* phase coincide with the falling *R*(*ω*) peaks of the B*_k_* phase at 20.8 and 24.9. In addition, the maximal *L*(*ω*) peak value (24.9) of the B*_i_* phase correlates closely with the *R*(*ω*) dropping peak points, i.e., 24.9. The expansion of the profile between 3 and 52 eV is seen to be nearly identical for both the B*_k_* and B*_i_* phases. Beyond 52 eV, the energy loss *L*(*ω*) is roughly zero. The energy loss function results also deduce that the B*_i_* phase is a transparent material in the UV region.

Based on the above results, it is anticipated that the B*_i_* phase possesses application-oriented electronic and optical characteristics. The wide bandgap energy and imaginary part of dielectric function *ε*_2_(ω) results infer that Bi-phase ZnO may be used to develop UV lasers, UV detectors, and high-frequency devices [2]. The *ε*_1_(ω) results in visible and ultraviolet regions express that the B*_i_* phase has applications in developing ZnO-based piezoelectric devices [57], MSM photosensors, and detectors [58,59]. In addition, absorption coefficient values of the B*_i_* phase in the UV region are the foundation for developing ZnO-based solar filters and UV optoelectronic devices [60]. Moreover, on the basis of the UV transparent nature found through the refractive index and loss function results, the B*_i_* phase may be used in developing ZnO-based electron transparent layers (ETLs) for perovskite solar cells (PSCs) [61] and transparent electrodes for photovoltaic cells and LEDs [62].

## 4. Conclusions

This study is conducted in the framework of DFT to predict the stability, electronic structure, phase transition, and optical characteristics of B*_i_*-type ZnO under stress. The first-principles LDA and hybrid functional B3LYP approach were used for geometry optimization and to examine the properties. B*_k_*-phase ZnO is found to undergo a phase transition to the B*_i_* phase at 16.1 GPa, which is consistent with the previous prediction. The band structure and DOS exploration have validated the wider and direct bandgap energy of the B*_i_* phase under pressure. A blue shift (increase) in bandgap is observed by increasing pressure, which leads to inducing novel optical characteristics. The optical investigation of the dielectric function shows that the B*_i_* phase exhibits a dielectric nature in the visible energy region and metallic behavior in the ultraviolet region. There is a small absorption coefficient in the infrared and visible regions and a high absorption peak in the ultraviolet region. The energy loss function and refractive index calculation have confirmed the transparent nature of the B*_i_* phase in the UV range. The results from the current investigations confirm the presence of the B*_i_* phase in the ZnO system, and its optoelectronic properties are provided. Furthermore, this opens up the possibility of using the B*_i_* phase in developing ZnO-based optoelectronic and photovoltaic devices.

## Figures and Tables

**Table 1:** The estimated lattice constraints, bond lengths, and cell volumes for B*_k_*_-_ and B*_i_*-type ZnO at 0 GPa and phase transition pressure 16.1 GPa. The present calculated values are compared with the previous research findings.

Pressure (GPa)	Phase	Space Group	Reference	a = b (Å)	c (Å)	Bond Angle *α* = *β*	*γ*	Bond Length (Å)	Volume (Å^3^)
0	B*_k_*	P63MC	Theory ^a^	3.397	5.132	90°	120°	-	-
			Theory ^b^	3.161	5.165	90°	120°	-	22.36
			Present	3.357	4.360	90°	120°	2.180	21.287
	B*_i_*	P-3M1	Theory ^b^	2.93	4.97	90°	120°	-	18.76
			Present	2.920	5.024	90°	120°	2.102	18.557
16.1	B*_k_*	P63MC	Present	3.290	4.220	90°	120°	2.11	19.785
	B*_i_*	P-3M1	Present	2.851	4.949	90°	120°	2.059	17.721

^a^ [33] ^b^ [36].

## Data Availability

Not applicable.
